# Sex− and species−biased gene flow in a spotted eagle hybrid zone

**DOI:** 10.1186/1471-2148-11-100

**Published:** 2011-04-14

**Authors:** Niclas Backström, Ülo Väli

**Affiliations:** 1 Department of Organismic and Evolutionary Biology, Museum of Comparative Zoology, Harvard University, 26 Oxford Street, Cambridge, MA 02138, USA; 2 Institute of Agricultural and Environmental Sciences, Estonian University of Life Sciences, Riia 181, 51014 Tartu, Estonia

## Abstract

**Background:**

Recent theoretical and empirical work points toward a significant role for sex-chromosome linked genes in the evolution of traits that induce reproductive isolation and for traits that evolve under influence of sexual selection. Empirical studies including recently diverged (Pleistocene), short-lived avian species pairs with short generation times have found that introgression occurs on the autosomes but not on the Z-chromosome. Here we study genetic differentiation and gene flow in the long-lived greater spotted eagle (*Aquila clanga*) and lesser spotted eagle (*A. pomarina*), two species with comparatively long generation times.

**Results:**

Our data suggest that there is a directional bias in migration rates between hybridizing spotted eagles in eastern Europe. We find that a model including post divergence gene flow fits our data best for both autosomal and Z-chromosome linked loci but, for the Z-chromosome, the rate is reduced in the direction from *A. pomarina *to *A. clanga*.

**Conclusions:**

The fact that some introgression still occurs on the Z-chromosome between these species suggests that the differentiation process is in a more premature phase in our study system than in previously studied avian species pairs and that could be explained by a shorter divergence time and/or a longer average generation time in the spotted eagles. The results are in agreement with field observations and provide further insight into the role of sex-linked loci for the build-up of barriers to gene flow among diverging populations and species.

## Background

To get deeper insight into the mechanisms behind population differentiation and speciation, a crucial step is to understand the genetic underpinnings of reproductive isolation. Earlier efforts have revealed that, given the proportion of the genome covered by the physical size of sex-chromosomes, loci located on these chromosomes may contribute appreciably more than expected to reduced fitness in hybrids [1]. Most of the evidence spring from analyses of species with male heterogamety (XY-systems), where advanced genetic or genomic tools have been available for some time. It has for example been shown that regions on the X-chromosome affect hybrid fitness in *Drosophila *crosses or introgression lines [2-4], that several genes that cause hybrid breakdown or hybrid sterility in *Mus *species map to the X-chromosome [5-8], and also that the relative size of the X-chromosome compared to the autosomes affects the rate whereby reproductive isolation evolves in *Drosophila *[9]. Recently, there has been an accumulation of evidence that points to that the Z-chromosome plays a correspondingly important role in organisms with female heterogamety (ZW-systems), for example birds and butterflies. These data are mostly based on other methods than species crosses and introgression lines but include observations to suggest that Z-linked loci are involved in species recognition traits, like coloration [10,11], and female mate choice preference [10,12,13], as well as in determining hybrid viability and sterility [11,14].

The reasons for why sex-chromosome linkage may be important for genes involved in reproductive isolation are manifold. Sex-linkage enables recessive alleles to be expressed in the heterogametic sex, enhancing the effects of epistatic interactions involving sex-linked alleles. This phenomenon is known as Haldane's rule [15,16], and the rule is a commonly applied explanation to why hybrids of the heterogametic sex suffer more severe fitness reduction in interspecific crosses. Sex-linkage may also facilitate the evolution of sex-specific or sexually antagonistic traits and similar to the 'fast X-effect' occasionally (but not ubiquitously) observed in mammals [17], the Z-chromosome evolves faster than the autosomes in birds [18,19]. In female heterogametic systems in particular, sex-linkage may enhance the efficacy of sexual selection since the Z-chromosome is inherited directly from father to son [20,21], and sex-linked loci evolving under sexual selection might cause more rapid advancements in the build-up of reproductive isolation than if the loci would have been autosomal [22]. Furthermore, although sex-linkage does not imply a complete lack of recombination and unless there are strong sex-biases in the rate of recombination, it is expected that the sex-chromosomes would have a reduced recombination rate compared to autosomes of similar size since the sex-chromosome only recombines in one sex. This could facilitate the diversifying effects of reinforcement if both a trait locus and the preference for that trait are sex-linked [23], as has been found in some species [10,13]. Comparing patterns of genetic differentiation and introgression between genomic regions, for example between autosomes and sex-chromosomes, is therefore an attractive means to get deeper insight into which regions that drive reproductive isolation between species of interest.

The greater spotted eagle *Aquila clanga *and the lesser spotted eagle *A. pomarina *are two closely related, partially sympatric, Eurasian birds of prey whose ranges overlap in eastern Europe. There is no complete reproductive barrier between the species and extensive interbreeding, prevalently between *A. pomarina *males and *A. clanga *females, has been detected across the contact zone [24]. The hybridization is introgressive, and although gene flow occurs in both directions [24], the introgression rate of nuclear markers (AFLPs) has been estimated to be about ten times higher than for mtDNA which, in agreement with Haldane's rule, possibly reflects a lower fitness of hybrid females compared to hybrid males [25].

Here we use a large subset of previously developed gene-based sequences [26-28], to investigate patterns of genetic differentiation and to quantify gene flow on the autosomes and on the Z-chromosome in a spotted eagle hybrid zone. As far as we are aware of this is one of very few studies of gene flow in species that are long-lived and that have long average generation times. The results show biases both between chromosome classes and between species and further strengthen the idea that sex-linked loci might play an important role in the build-up of reproductive isolation between species. However, while previous studies on short-lived avian species pairs with short generation times have found evidence for a very reduced, or even complete absence of gene flow on the sex-chromosomes [11,29,30], we still find some introgression on the Z-chromosome between *A. pomarina *and *A. clanga*.

## Methods

### Study species

**Figure 1:**
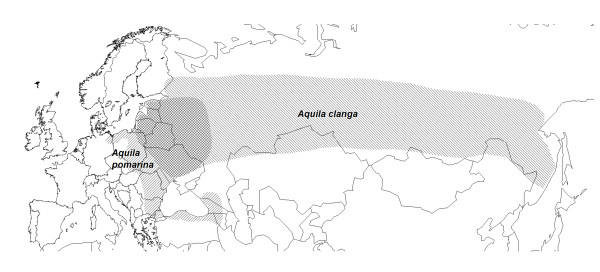
**Distribution ranges**. Global distribution ranges of the greater spotted eagle and the lesser spotted eagle. The hybrid zone is in the area where the species ranges overlap.

The spotted eagles are medium-sized long-lived raptors with a generation time of approximately 11 years [31,32]. These monogamous birds form sparsely distributed solitary pairs and use the same nest over consecutive years [33]. Both species inhabit mosaic landscapes: they nest in forest and hunt in open landscape [33]. Although the preferred habitats differ somewhat between the species, *A. clanga *being more closely associated to water, the overlapping distribution ranges (Figure 1), partly similar habitat usage and behavioral similarities still makes interbreeding possible [34].

The numbers of both species have decreased during the last century [35], and the decline has been particularly dramatic in *A. clanga*, whose vast range across Eurasia is occupied only by few thousand pairs with less than thousand pairs breeding in Europe [31]. In contrast, populations of *A. pomarina *are still dense, and in most regions significantly outnumber the sparsely represented *A. clanga *[31,35]. Both species are listed in Annex I of the EU Directive on the Conservation of Wild Birds (EEC/79/409), as well in the IUCN Red List: *A. clanga *as a globally vulnerable species and *A. pomarina *as a species of least conservation concern [36].

### Sampling and DNA extraction

We studied 14 unrelated individuals (seven *A. clanga*, seven *A. pomarina*; three males and four females from each species). These were collected in the sympatric region in eastern Europe; 13 in Estonia and one in Poland (Figure 1). Autosomal markers were analyzed in five birds from each of the species, but in the analysis of Z-chromosomal markers two more birds were added in order to obtain the same number of chromosomes. Hence, for both autosomal and Z-chromosomal loci, 10 chromosomes were analyzed in each of the species

Blood samples were collected from nestlings or from trapped adults. DNA was extracted from blood cells using proteinase K treatment followed by a salting [37], or a phenol-chloroform purification method [38]. Species-specific morphological characters [39,40] were investigated carefully and used for species assignment. The final pre-assignment was made, however, using 9 diagnostic SNPs and 20 microsatellites [41] together with a large number of comparative samples, within an extensive pan-European hybridization survey [24]. All individuals were assigned to either of the species with a high probablility (> 90%) by two Bayesian model-based assignment methods [42,43].

### Marker development

We used a large subset of previously developed gene-based sequences [26-28]. First, we tested amplification of 122 autosomal and 50 Z-linked primer pairs using a single high-quality *A. clanga *sample, and obtained 87 and 21 single-band amplification products, respectively, in other cases we recorded either several products or no DNA-band at all. Strongest single-band PCR products were selected for sequencing resulting in high-quality sequence data for 36 autosomal and 15 Z-chromosomal loci to use in further analysis. All sequences included in this study have been submitted to GenBank under accession numbers JF521998 - JF522099.

Amplification was performed in 25 μl containing 25-50 ng DNA, 0.25 U AmpliTaq Gold polymerase with 1 × Amplitaq Gold PCR buffer (Applied Biosystems) or Hotstar Taq polymerase with 1 × buffer (Qiagen), 2.5 mM MgCl2, 0.5 μM of each primer and 0.2 mM dNTP. The PCR profile included an initial heating at 95°C for 5 min, followed by 35 cycles of 95°C for 30 s, 60°C to 50°C for 30 s and 72°C for 1 min, and a final extension at 72°C for 10 min. During first subset of cycles (10 or 20), an annealing temperature was decreased by 0.5°C or 1°C for every cycle, whereas for the remaining cycles 50°C was used.

PCR fragments for sequencing were purified by exonuclease I and shrimp alkaline phosphatase (USB) treatment at 37°C for 15 min, followed by denaturation at 80°C for 15 min. Sequencing was performed by DYEnamic ET Terminator or BigDye Terminator sequencing reagent premix and MegaBACE 1000 or ABI 3750 automated capillary sequencer (Amersham Biosciences) according to the manufacturer's recommendations. The PCR products were purified in AutoSeq96 plates or using an X-terminator purification kit (both by Amersham Biosciences).

### Data analysis

All sequences were edited in Sequencher (Gene Codes Corp.) and aligned with Clustal W [44] as implemented in MEGA4 [45]. Using information of intron-exon boundaries from the orthologous genes in chicken, sequences were cut to only include the intronic part of the gene. All sequences were subsequently purged for simple sequence motifs with the help of Sputnik http://espressosoftware.com/sputnik/index.html and individual haplotypes were resolved with PHASE v2.1 [46,47]. For all loci separately we calculated the population genetic summary statistics number of segregating sites (S), nucleotide diversity (π), Tajima's *D *(Tajima 1989), *F_ST _*and minimum number of recombination events (R) using DnaSP [48] (Additional file 1).

A six-parameter isolation-migration model (IM) [49,50] was applied to the data to get estimates of the level of gene flow for the different chromosome classes. The output of IM contains Maximum Likelihood estimates and the posterior probability distributions for the parameters Θ_1 _(4N_e1_μ, population mutation rate for population 1, N_e _= effective population size), Θ_2 _(4N_e2_μ, population mutation rate for population 2), Θ_A _(4N_eA_μ, population mutation rate for the ancestral population), τ (tμ, time since divergence), m_1 _(m_1_/μ, migration rate from population 2 to population 1 when looking forward in time) and m_2 _(m_2_/μ, migration rate from population 1 to population 2 when looking forward in time). The model assumes neutrally evolving loci and, complementary to the analysis of allele frequency distributions (Tajima's *D *[51], see above), we therefore applied a multi-locus HKA-test [52], as implemented by the software HKA http://genfaculty.rutgers.edu/hey/software#HKA, and a Bayesian method (BAYESFST) [53] to investigate if any locus showed evidence for directional or balancing selection. Selection tests were applied to the autosomal and the Z-linked loci, separately. No locus showed evidence for selection at the 1% confidence level and all loci where therefore assumed to evolve neutrally and used in subsequent demographic analysis. Inter-specific levels of differentiation (*F_ST_*, as calculated in DnaSP [48]) and the corresponding untransformed p-values for each locus, as calculated in BAYESTFST [53], are given in the Additional file 1. A second major assumption of IM is that there should be no intra-locus recombination [49]. Therefore we applied the four-gamete-test (minimum number of recombination events > 0) [54] as implemented in DnaSP [48] to each locus. Only 6% (3/51) of the loci showed signs of recombination after applying the four-gamete-test and these were cut so that the longest sequence without evidence for recombination was analyzed.

**Figure 2:**
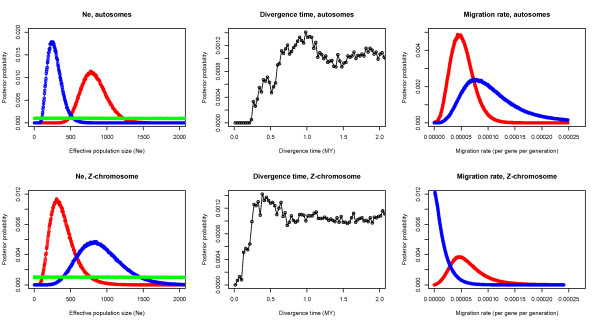
**Posterior probability distributions for the IM model**. Posterior probability distributions for the full Isolation-Migration model for autosomal and Z-linked loci, respectively. The distribution from the longest (100 Million steps) run is plotted for each chromosomal class. In the plots of the effective population size (N_e_), red = *A. clanga*, blue = *A. pomarina *and green = ancestral population. In the plots of the migration rates, red = migration from *A. clanga *to *A. pomarina *and blue = migration rate from *A. pomarina *to *A. clanga*.

In the IM runs, autosomal and Z-linked loci were analyzed separately and each class was run with identical settings for three independent runs but with different random seeds. Each dataset was run with a wide prior parameter range (q (Θ = 4N_e_μ for each population) = 0-10, m (m/μ for each direction) = 0-50, t (tμ) = 0 - 50) in an initial run with a burn-in of 5*10^5 ^followed by 5*10^6 ^steps and the posterior estimates for the parameters from this initial run was used to narrow the priors (q (Θ = 4N_e_μ for each population) = 0-2, m (m/μ for each direction) = 0-25, t (tμ) = 0 - 25) in two subsequent, longer analyses. These were run for 5*10^7 ^and 1*10^8 ^cycles with a burn-in of 1*10^6 ^steps, respectively. There was good agreement in parameter posterior probability distributions and maximum likelihood estimates between independent runs and therefore we only report the values from the longest run for autosomal and Z-chromosome data, respectively (Figure 2, Table 1). The mixing of chains was satisfactory and in most runs all Effective Sample Size (ESS) values exceeded 2000 and for no parameter was the ESS lower than 333 (Table 1). All six parameter values were scaled with a per site per year mutation rate (μ) of 1.4*10^-9 ^[55] and a generation time of 11 years [31,32]. The different inheritance modes for autosomes (1) and the Z-chromosome (3/4) were accounted for by setting their respective inheritance scalars in the IM input file. For details about the parameter calculations, see the introduction to IM documentation http://genfaculty.rutgers.edu/hey/software#IM.

**Table 1:** Summary of posterior values from the IM analysis

	*A. cla *N_e_	*A. pom *N_e_	Anc. N_e_	t	*A. cla > A. pom*	*A. pom > A. cla*	*» A. pom*	*» A. cla*
**Autosomes**								
HiPt	8595	2617	448	983	3.9	6.8	0.74	0.39
LHPD90	5862	1364	78	623	1.6	2.8	0.21	0.083
HHPD90	12119	4721	52389	27672	7.6	16.3	2.0	1.7
ESS	8282	3175	38954	333	5111	3027		
**Z-chromosome**								
HiPt	3279	9071	65	374	4.5	0.034	0.32	0.0068
LHPD90	1412	4483	65	235	1.3	0.0011	0.040	0.0011
HHPD90	6884	14688	43406	27672	10.4	3.4	1.6	1.09
ESS	9201	12030	39997	2517	9431	6606		

Table 1 legend: Summary of posterior values from the IM analysis scaled by a per gene per generation mutation rate of 1.4*10^-9^. Given is the highest point estimate for each parameter (HiPt), the lower (LHPD90) and higher (HHPD90) boundaries for the 90% highest posterior density and the effective sample sizes for each parameter (ESS). Only values from the two longest (1 Million steps burn-in + 100 Million steps run time) runs are tabulated. Per generation population migration rates into (») each species is also given. *A. cla *= *Aquila clanga, A. pom = Aquila pomarina*, Anc. = ancestral population, N_e _= effective population size, > = gene flow direction (per gene per generation *10^-6^), t = time since divergence (1000 years).

Finally, we compared the model that included all six parameters (Θ_1_, Θ_2_, Θ_A_, t, m_1 _and m_2_), to a simpler demographic model that did not allow for post population divergence gene flow (m_1 _= m_2 _= 0). These analyses were conducted using IMa [56] by running an initial M-mode run with the identical settings to the IM runs (full 6-parameter model) and sampling 3*10^5 ^genealogies followed by a subsequent L-mode run analyzing all possible nested models. The significance of the difference between models was evaluated by applying likelihood ratio tests as implemented in the software.

## Results

### General, levels of polymorphism and signs of selection

We sequenced 36 autosomal (23.2 kb in total) and 15 Z-linked (9.4 kb) genes in 10 individuals from each species. In total, we found 97 single nucleotide polymorphisms (SNPs) in *A. clanga *and 79 SNPs in *A. pomarina *and 52 of these were shared between the species. Only four fixed differences were detected and three of these were located on the Z-chromosome. None of the genes showed evidence for selection, neither in the HKA test (Sum of deviations = 39.5, DF = 100, P = 0.99), in the allele frequency distributions (Tajima's *D*), or in the Bayesian analysis after correcting for multiple tests. The average nucleotide diversity was higher on the autosomes than on the Z-chromosome in both *A. clanga *(autosomes = (mean) 1.3*10^-3 ^± (SD) 3.8*10^-4^; Z-chromosome = 7.4*10^-4 ^± 3.2*10^-4^) and *A. pomarina *(autosomes = 9.7*10^-4 ^± 3.3*10^-4^; Z-chromosome = 7.6*10^-4 ^± 2.8*10^-4^), however, the difference was minor in *A. pomarina *(Wilcoxon's test, *W *= 305, P = 0.47), and only close to significant in *A. clanga *(*W *= 358, P = 0.069). As can be read from the figures above, the average autosomal nucleotide diversity was higher in *A. clanga *(1.3*10^-3^) than in *A. pomarina *(9.7*10^-4^), however this difference was not significant (*W *= 767, P = 0.18), and there was no significant difference between the species for the Z-chromosome (7.4*10^-4 ^in *A. clanga *and 7.6*10^-4 ^in *A. pomarina*, Wilcoxon's test, *W *= 102, P = 0.66).

### Genetic differentiation among species

The overall *F_ST _*between *A. clanga *and *A. pomarina *was 0.30 ± 0.29. The level of differentiation was higher for the Z-chromosome (0.37 ± 0.38) than for the autosomes (0.27 ± 0.25), but the variance was also higher on the Z-chromosome and the difference between chromosome classes was not significant (*W *= 253, P = 0.73).

### Gene flow between species

We ran IMa analyses for autosomal and Z-chromosome data separately and evaluated the significance of a model with free and independent gene flow in both directions to different nested models with restricted gene flow (Table 2). For autosomal data, the model with gene flow in both directions was significantly better than all models with restricted gene flow in any or both directions (2*log likelihood ratio (LLR) range = 5.01 - 920.34, df = 1-2, P range = < 1.0*10^-6 ^- 0.025). However, the model with unequal gene flow between species was not significantly better than the model with similar gene flow in both directions (2*log likelihood ratio = 1.19, df = 1, P = 0.27). For Z-chromosome data, the model with gene flow in both directions was significantly better than a model without any gene flow (2*LLR = 235.39, df = 2, P < 1.0*10^-6^) and a model with no gene flow from *A. clanga *to *A. pomarina *(2*LLR = 11.2, df = 1, P = 8.0*10^-4^) but not better than a model with no gene flow from *A. pomarina *to *A. clanga *(2*LLR = 0.0048, df = 1, P = 0.94).

**Table 2:** Summary of likelihood ratio test statistics for the IMa analysis

Model	log (P)	2LLR	df	P-value
**Autosomes**				
ABC0D (m1 = 0)	-2.85	5.01	1	0.025 *
ABCD0 (m2 = 0)	-171.40	342.11	1	< 1.0*10^-6 ^***
ABC00 (m1 = m2 = 0)	-460.52	920.34	2	< 1.0*10^-6 ^***
ABCDD (m1 = m2 > 0)	-0.94	1.19	1	0.27
**Z-chromosome**				
ABC0D (m1 = 0)	-6.12	11.20	1	8.0*10^-4 ^***
ABCD0 (m2 = 0)	-0.52	0.0048	1	0.94
ABC00 (m1 = m2 = 0)	-118.21	235.39	2	< 1.0*10^-6 ^***
ABCDD (m1 = m2 > 0)	-0.90	0.76	1	0.37

Table 2 legend: Summary of likelihood ratio statistics (2LLR), degrees of freedom (df) and significance level (P-value) when comparing the full likelihood model (ABCDE) to four different nested models (ABC0D (m1 = 0), ABCD0 (m2 = 0), ABC00 (m1 = m2 = 0), ABCDD (m1 = m2 > 0)), using IMa for autosomal and Z-linked loci, respectively.

The full isolation migration model (IM) revealed biases in the patterns of gene flow between species and among chromosome classes. For autosomal loci there was a marginally higher degree of gene flow from *A. pomarina *to *A. clanga *(HiPt = 6.8*10^-6^) than from *A. clanga *to *A. pomarina *(HiPt = 3.9*10^-6^) (Figure 2, Table 1). The proportion of genealogies where this direction was inferred was 0.81. For the Z-chromosomal loci, the estimated amount of gene flow was similar to the autosomal rate in the direction from *A. clanga *to *A. pomarina *(HiPt = 4.5*10^-6^), but the rate was severely reduced in the direction from *A. pomarina *to *A. clanga *(HiPt = 3.4*10^-8^) (Figure 2, Table 1). The proportion of genealogies where the rate from *A. clanga *to *A. pomarina *was higher than the rate from *A. pomarina *to *A. clanga *was 0.93. These patterns are also evident from the scaled population migration rate estimates, which were higher for the autosomal loci than for the Z-linked loci into both species and drastically reduced into *A. clanga *for the Z-linked loci (Table 1). For most parameters in most runs we got good convergence and the posterior probability tails were zero within the parameter range. However, in some runs, the posterior probability tails for ancestral population size and the time since divergence were still increasing at the highest boundary of the parameter interval.

## Discussion

We analysed 36 autosomal and 15 Z-linked introns in population samples of greater spotted eagles and lesser spotted eagles from a hybrid zone in eastern Europe. The observed levels of genetic diversity were lower for Z-linked than for autosomal loci in both species. Since the Z-chromosome has a lower population size than autosomes, the Z:A population size ratio would be 3:4 in a population with equal amounts of reproducing males and females, which is probably a valid assumption in these monogamous species. Hence, the expected diversity of the Z-chromosome would be approximately 75% of the diversity observed for autosomes. This is in good agreement with the diversity level observed on the *A. pomarina *Z-chromosome (78% of the autosomal diversity level), but the diversity is lower than expected for the Z-chromosome in *A. clanga *(57%). It should be noted that the effect of a potential male mutation bias would result in an increase of the Z:A diversity ratio. Given the limited difference in mutation rate between chromosomal classes in birds this should probably only have a relatively small effect on the diversity levels [55,57], but still suggests that the observed Z-chromosome diversities are at least not higher than expected, especially in *A. clanga*. It has been suggested that selection might be more intense on the Z-chromosome than on the autosomes and that recurrent selective sweeps therefore can cause a reduction in diversity below the level expected from differences in population size and mutation rates only [58]. Recent data indicate that selective sweeps affect the diversity levels only at very limited distances from the selected site [e.g. [59,60]], and it might be unlikely to expect selective sweeps to occur frequently enough to reduce diversity levels over the entire chromosome.

Analogous to the loss of genetic diversity for chromosomes with smaller effective population sizes (see paragraph above), genes on the Z-chromosome are expected to accumulate allele frequency differences and fixed substitutions between diverging populations more rapidly than genes on autosomes. We did not find evidence for this in the spotted eagles. The level of differentiation was on average higher for Z-linked genes than for autosomal genes. However, the variance among genes was also higher for Z-linked than for autosomal genes and the difference between chromosomal classes was not significant.

Previous work on speciation genetics has pointed out that genomic regions harboring genes that affect reproductive isolation (so called 'genomic islands of speciation') should experience lower rates of interspecific recombination than the genome in general [61,62]. These islands may subsequently act as drivers of isolation through epistatic or physical interactions to other loci in the genome resulting in expansion of non-recombining regions and, at the end, complete reproductive isolation [63], although some regions may stay porous to post divergence gene flow for significant amounts of time [64]. Preliminary attempts using a few loci points towards regional variation in degree of interspecific recombination among sex-linked genes in *Passerina *buntings [65], and more generally, recurrent analyses involving several bird species pairs have revealed that the rate of introgression is significantly reduced on the Z-chromosome compared to the autosomes. When studying multi-locus SNP data in the pied flycatcher (*Ficedula hypoleuca*) and the collared flycatcher (*F. albicollis*), Sætre et al. [11] found no evidence for introgression on the Z-chromosome despite frequent introgression on the autosomes. Similarly, Storchová et al. [30] analyzed interspecific migration rates between closely related nightingale species (*Luscinia luscinia *and *L. megarhynchos*) and found that gene flow occurred on the autosomes but was completely absent from the Z-chromosome. Additionally, Carling et al. [29] found evidence for autosomal gene flow subsequent to the initial divergence of the Lazuli bunting (*Passerina amoena*) and the indigo bunting (*P. cyanea*) but they could not reject a strict allopatric model of divergence when analyzing Z-chromosome linked loci. In agreement with abovementioned studies our analyses showed that introgression rates were lower on the Z-chromosome than on the autosomes from *A. pomarina *to *A. clanga*. In fact we could only reject a model without post-divergence gene flow from *A. pomarina *to *A. clanga *for autosomal genes, not for Z-linked genes. There was no reduction in introgression on the Z-chromosome compared to the autosomes from *A. clanga *to *A. pomarina *and we could reject the model without post-divergence gene flow, indicating that gene flow still occurs on the Z-chromosome in that direction.

Hence, our data suggest that there has been post-divergence gene flow in both directions for the autosomes, but predominantly from *A. clanga *to *A. pomarina*, for Z-chromosome linked genes. There are several ways in which these results could be interpreted. In agreement with the dominance theory [66,67] of Haldane's rule [15], it could be that the reduced Z-chromosome introgression from *A. pomarina *to *A. clanga *is a result of incompatibilities between autosomal alleles specific to *A. clanga *and Z-chromosome alleles specific to *A. pomarina*, and less severely between Z-chromosome alleles specific to *A. clanga *and autosomal alleles specific to *A. pomarina*. A perhaps equally likely and not necessarily mutually exclusive explanation is that there are sex-biases in hybridization rates between the species. It is known from field observations that interbreeding usually occurs between *A. pomarina *males and *A. clanga *females whereas *A. clanga *males interbreed with *A. pomarina *females less frequently [24]. This is supported by mitochondrial data which suggests that mtDNA is introgressing into *A. pomarina *[25], and by autosomal data indicating backcrossing mostly to *A. pomarina *[24]. If hybridization only occurs between *A. clanga *females and *A. pomarina *males and if hybrid females have low fitness as data suggest [25], it is possible that gene flow on the Z-chromosome would be restricted to only occur from *A. clanga *to *A. pomarina*. This uni-directional gene flow could potentially also explain the comparatively high diversity levels observed for Z-linked genes in *A. pomarina *(i.e. introgressed *A. clanga *alleles contributing to higher diversity).

The divergence time between *A. clanga *and *A. pomarina *has been estimated to be approximately one million years [68]. This is roughly similar to comparisons between other avian species pairs where Z-chromosome introgression seems to be reduced or completely absent [11,29,30,69]. Caution should be taken, however, since divergence time estimates that apply a molecular clock using few loci might deviate significantly from estimates based on likelihood analysis applying an isolation migration model [e.g. [70]]. The divergence time between *A. clanga *and *A. pomarina *is based on mitochondrial divergence [68] whereas the estimates for both the *Luscinia *nightingales and the *Passerina *buntings are based on multi-locus data and the divergence for the mitochondria is considerably deeper for both of these species [29,30]. A possible explanation for the low degree of reproductive isolation between the spotted eagles compared to previously studied avian taxa is therefore simply that the divergence time is significantly shorter for these species. However, the notably longer life span and generation times of eagles compared to passerines [31] could also have an effect. Hybrid incompatibilities are expected to evolve slowly [71,72], and the rate probably decreases with increasing life-span and generation times due to maintenance of ancestral polymorphisms [e.g. [73]].

## Conclusions

We studied gene flow, genetic differentiation and genetic diversity for 36 autosomal and 15 Z-linked genes in a spotted eagle hybrid zone in eastern Europe. Our data suggest that introgression occurs in both directions but that introgression is reduced on the Z-chromosome compared to the autosomes from *A. pomarina *to *A. clanga*. This is one of few studies analyzing long-lived bird species and we show that the barriers in the spotted eagles are more permeable to gene flow than previously studied avian species pairs. The data support an important role for sex-linked loci in the build-up of barriers to gene flow and supports a model where reproductive barriers evolve in a step-wise manner rather than instantly. The observation of reduced gene flow on the Z-chromosome together with data that indicate a larger variance in level of differentiation between sex-linked than between autosomal loci makes it tempting to suggest that interspecific recombination maintains shared alleles at some sex-linked loci while more rapid accumulation of fixed differences occurs at loci with restricted interspecific recombination. The latter could constitute potential 'genomic islands of speciation' and are obvious targets for subsequent efforts aiming at identifying loci that contribute to reproductive isolation between these species.

## Authors' contributions

ÜV did all fieldwork, analysis of specimen morphology and the molecular genetic work. NB carried out all sequence analyses and drafted the manuscript. Both authors read and approved the final manuscript.

## Supplementary Material

Additional file 1 **DNA sequence summary statistics for all loci included in the study**. Summary statistics from the sequence data for 36 autosomal and 15 Z-chromosome linked genes. Chr = chromosome (in chicken), Gene = gene name for the ortholog in chicken or, when no data were at hand for chicken, for another vertebrate species, *A. cla *= *Aquila clanga *(greater spotted eagle), *A. pom *= *Aquila pomarina *(lesser spotted eagle), π = nucleotide diversity, S = number of segregating sites, Fix = number of fixed differences between the species, Share = number of shared polymorphisms between the species, *D *= Tajima's *D *statistic, *F_ST _*= Hudson's *F_ST_*, p-val = untransformed p-value from the BAYESFST analysis. Click here for file
