# Supplementary material for: Sex− and species−biased gene flow in a spotted eagle hybrid zone
Source: BMC Evol Biol. 2011 Apr 14;11:100. doi: 10.1186/1471-2148-11-100 (PMC3098175; doi:10.1186/1471-2148-11-100)
Supplement: Additional file 1 — DNA sequence summary statistics for all loci included in the study. Summary statistics from the sequence data for 36 autosomal and 15 Z-chromosome linked genes. Chr = chromosome (in chicken), Gene = gene name for the ortholog in chicken or, when no data were at hand for chicken, for another vertebrate species, A. cla = Aquila clanga (greater spotted eagle), A. pom = Aquila pomarina (lesser spotted eagle), π = nucleotide diversity, S = number of segregating sites, Fix = number of fixed differences between the species, Share = number of shared polymorphisms between the species, D = Tajima's D statistic, FST = Hudson's FST, p-val = untransformed p-value from the BAYESFST analysis. [file 1471-2148-11-100-S1.DOC]

**Additional file.** Summary statistics from the sequence data for 36 autosomal and 15 Z-chromosome linked genes. Chr = chromosome (in chicken), Gene = gene name for the ortholog in chicken or, when no data were at hand for chicken, for another vertebrate species, *A. cla* = *Aquila clanga* (greater spotted eagle), *A. pom* = *Aquila pomarina* (lesser spotted eagle), π = nucleotide diversity, S = number of segregating sites, Fix = number of fixed differences between the species, Share = number of shared polymorphisms between the species, *D* = Tajima’s *D* statistic, *F_ST_* = Hudson’s *F_ST_*, p-val = untransformed p-value from the BAYESFST analysis.

| **Chr** | **Gene** | **Length** | ***A. cla* π** | ***A. pom* π** | ***A. cla* S** | ***A. pom* S** | **Fix** | **Share** | ***A. cla* *D*** | ***A. pom D*** | ***F_ST_*** | **p-val** |
| --- | --- | --- | --- | --- | --- | --- | --- | --- | --- | --- | --- | --- |
| 1 | *IGFBP7* | 277 | 0.00257 | 0.00000 | 2 | 0 | 0 | 0 | 0.019 | na | 0.111 | 0.243 |
| 1 | *ATP6V1* | 484 | 0.00073 | 0.00000 | 1 | 0 | 0 | 0 | 0.015 | na | 0.111 | 0.409 |
| 1 | *ACP6* | 807 | 0.00083 | 0.00044 | 2 | 1 | 0 | 1 | -0.184 | 0.015 | 0.290 | 0.506 |
| 1 | *ASMTL* | 562 | 0.00119 | 0.00134 | 2 | 2 | 0 | 2 | -0.184 | 0.222 | 0.387 | 0.631 |
| 1 | *UBAC2* | 434 | 0.00210 | 0.00210 | 2 | 3 | 0 | 2 | 0.932 | -0.507 | 0.340 | 0.761 |
| 2 | *ARF1* | 1076 | 0.00101 | 0.00052 | 3 | 2 | 0 | 1 | 0.096 | -0.691 | 0.144 | 0.719 |
| 2 | *YME1L1* | 873 | 0.00023 | 0.00076 | 1 | 2 | 0 | 1 | -1.112 | -0.184 | 0.097 | 0.746 |
| 2 | *PAK1IP* | 645 | 0.00103 | 0.00086 | 2 | 2 | 0 | 1 | -0.184 | -0.691 | 0.608 | 0.261 |
| 2 | *CHMP5* | 406 | 0.00705 | 0.00562 | 4 | 3 | 0 | 3 | 0.928 | 1.152 | 0.187 | 0.529 |
| 2 | *Unknown* | 467 | 0.00043 | 0.00114 | 1 | 1 | 0 | 1 | -1.112 | 1.303 | 0.368 | 0.518 |
| 3 | *IGF2R* | 383 | 0.00052 | 0.00000 | 1 | 0 | 0 | 0 | -1.112 | na | 0.000 | 0.431 |
| 3 | *UBC6* | 669 | 0.00030 | 0.00083 | 1 | 2 | 0 | 1 | -1.112 | -0.691 | 0.006 | 0.672 |
| 3 | *RNASEH1* | 509 | 0.00161 | 0.00218 | 2 | 2 | 0 | 2 | 0.242 | 1.621 | 0.036 | 0.760 |
| 4 | *ARHGEF9* | 745 | 0.00089 | 0.00095 | 2 | 2 | 0 | 2 | -0.184 | 0.019 | 0.493 | 0.365 |
| **Chr** | **Gene** | **Length** | ***A. cla* π** | ***A. pom* π** | ***A. cla* S** | ***A. pom* S** | **Fix** | **Share** | ***A. cla* *D*** | ***A. pom D*** | ***F_ST_*** | **p-val** |
| 4 | *ADH5* | 563 | 0.00063 | 0.00000 | 1 | 0 | 0 | 0 | 0.015 | na | 0.111 | 0.679 |
| 4 | *Unknown* | 444 | 0.00230 | 0.00045 | 3 | 1 | 1 | 0 | -0.130 | -1.112 | 0.641 | 0.394 |
| 5 | *Unknown* | 520 | 0.00038 | 0.00000 | 1 | 0 | 0 | 0 | -1.112 | na | 0.889 | 0.158 |
| 5 | *PSMC1* | 951 | 0.00095 | 0.00021 | 3 | 1 | 0 | 1 | -1.034 | -1.112 | 0.627 | 0.647 |
| 6 | *VPS26A* | 466 | 0.00238 | 0.00272 | 4 | 5 | 0 | 4 | -0.822 | -1.136 | -0.081 | 0.075 |
| 6 | *PDCD11* | 700 | 0.00213 | 0.00267 | 4 | 4 | 0 | 3 | -1.562 | 1.229 | 0.283 | 0.155 |
| 7 | *ACADL* | 960 | 0.00208 | 0.00111 | 4 | 3 | 0 | 3 | 1.591 | 0.021 | 0.248 | 0.895 |
| 7 | *BZW1* | 590 | 0.00094 | 0.00185 | 2 | 3 | 0 | 0 | -0.691 | 0.096 | 0.178 | 0.181 |
| 8 | *DDAH1* | 597 | 0.00067 | 0.00034 | 2 | 1 | 0 | 1 | -1.401 | -1.112 | -0.071 | 0.503 |
| 8 | *MAGOH* | 603 | 0.00383 | 0.00151 | 5 | 3 | 0 | 3 | 1.233 | -0.507 | 0.240 | 0.693 |
| 10 | *DUT1* | 475 | 0.00042 | 0.00075 | 1 | 1 | 0 | 1 | -1.112 | 0.015 | 0.625 | 0.610 |
| 10 | *EIF3J* | 1087 | 0.00033 | 0.00000 | 1 | 0 | 0 | 0 | 0.015 | na | 0.111 | 0.615 |
| 11 | *CSNK2A2* | 689 | 0.00161 | 0.00000 | 3 | 0 | 0 | 0 | 0.172 | na | 0.306 | 0.355 |
| 12 | *SLMAP* | 753 | 0.00000 | 0.00074 | 0 | 2 | 0 | 0 | na | -0.691 | 0.000 | 0.394 |
| 13 | *DPYSL3* | 1033 | 0.00039 | 0.00039 | 2 | 2 | 0 | 2 | -1.401 | -1.401 | 0.756 | 0.205 |
| 14 | *FAM18A* | 853 | 0.00065 | 0.00080 | 2 | 2 | 0 | 1 | -0.691 | -0.448 | 0.013 | 0.790 |
| 17 | *EDF1* | 631 | 0.00151 | 0.00032 | 3 | 1 | 0 | 1 | -0.356 | -1.112 | 0.593 | 0.267 |
| 23 | *RPL11* | 788 | 0.00116 | 0.00093 | 2 | 2 | 0 | 2 | 0.932 | 0.120 | 0.224 | 0.776 |
| 24 | *CRTAM* | 492 | 0.00000 | 0.00072 | 0 | 1 | 0 | 0 | na | 0.015 | 0.111 | 0.277 |
| 26 | *RPS10* | 461 | 0.00043 | 0.00159 | 1 | 2 | 0 | 1 | -1.112 | 0.120 | 0.314 | 0.700 |
| **Chr** | **Gene** | **Length** | ***A. cla* π** | ***A. pom* π** | ***A. cla* S** | ***A. pom* S** | **Fix** | **Share** | ***A. cla* *D*** | ***A. pom D*** | ***F_ST_*** | **p-val** |
| 27 | *DDX42* | 787 | 0.00045 | 0.00000 | 1 | 0 | 0 | 0 | 0.015 | na | 0.111 | 0.628 |
| 28 | *POLR2E* | 428 | 0.00362 | 0.00093 | 5 | 2 | 0 | 2 | 0.124 | -1.401 | 0.427 | 0.721 |
| Z | *NNT* | 682 | 0.00029 | 0.00081 | 1 | 2 | 0 | 0 | -1.112 | -0.691 | 0.685 | 0.152 |
| Z | *PARP8* | 587 | 0.00000 | 0.00091 | 0 | 1 | 0 | 0 | na | 1.303 | 0.333 | 0.493 |
| Z | *ITA1* | 708 | 0.00075 | 0.00000 | 1 | 0 | 1 | 0 | 1.303 | na | 0.810 | 0.541 |
| Z | *SV2C* | 658 | 0.00267 | 0.00061 | 5 | 2 | 0 | 2 | -0.027 | -1.401 | 0.527 | 0.774 |
| Z | *ASAH3L* | 690 | 0.00135 | 0.00148 | 2 | 2 | 0 | 2 | 1.033 | 1.439 | -0.063 | 0.022 |
| Z | *MLSN2* | 204 | 0.00000 | 0.00000 | 0 | 0 | 1 | 0 | na | na | 1.000 | 0.833 |
| Z | *TRPM6* | 937 | 0.00000 | 0.00050 | 0 | 1 | 0 | 0 | na | 0.820 | 0.222 | 0.608 |
| Z | *PSAT1* | 690 | 0.00000 | 0.00029 | 0 | 1 | 0 | 0 | na | -1.112 | 0.000 | 0.791 |
| Z | *UNKN4* | 366 | 0.00206 | 0.00243 | 3 | 2 | 0 | 2 | -1.034 | 0.830 | 0.106 | 0.306 |
| Z | *ABCA1* | 540 | 0.00000 | 0.00000 | 0 | 0 | 1 | 0 | na | na | 1.000 | 0.870 |
| Z | *IPO11* | 549 | 0.00166 | 0.00255 | 3 | 4 | 0 | 1 | -0.507 | -0.038 | 0.219 | 0.529 |
| Z | *TRP1* | 735 | 0.00048 | 0.00027 | 1 | 1 | 0 | 0 | 0.015 | -1.112 | 0.074 | 0.343 |
| Z | *UNKN2* | 835 | 0.00141 | 0.00073 | 4 | 3 | 0 | 3 | -1.030 | -1.562 | -0.046 | 0.292 |
| Z | *SNX30* | 443 | 0.00000 | 0.00060 | 0 | 1 | 0 | 0 | na | -1.112 | 0.000 | 0.378 |
| Z | *UNKN7* | 757 | 0.00047 | 0.00026 | 1 | 1 | 0 | 0 | 0.015 | -1.112 | 0.691 | 0.065 |
